# An aggressive soft-tissue sarcoma of the extremity: A myxofibrosarcoma, grade 3 (FNCLCC system)

**DOI:** 10.1016/j.radcr.2024.03.092

**Published:** 2024-05-03

**Authors:** Manuela Montatore, Federica Masino, Gianmichele Muscatella, Rossella Gifuni, Giacomo Fascia, Alessio Sciacqua, Giuseppe Guglielmi

**Affiliations:** a Department of Clinical and Experimental Medicine, Foggia University School of Medicine, Viale L. Pinto 1, 71122 Foggia, (FG) Italy; b Radiology Unit, “Dimiccoli” Hospital, Viale Ippocrate 15, 70051, Barletta (BT), Italy; c Radiology Unit, “IRCCS Casa Sollievo della Sofferenza” Hospital, Viale Cappuccini 1,71013 San Giovanni Rotondo, (FG) Italy

**Keywords:** Sarcomas, STS, Soft tissue sarcoma, Soft tissue neoplasm, Myxofibrosarcoma, MFH, Extremity sarcoma, Diagnostic procedure, CT, MRI

## Abstract

We report a case of myxofibrosarcoma of the posterior region of the femur, part of the group of soft-tissue sarcomas: a set of rare and heterogeneous tumors with various subtypes and different prognostic. It is characterized by local infiltrative activity and an extremely high rate of local recurrence. A 58-year-old man came to the Radiology Department to examine a voluminous round and expansive formation of the posterior thigh region. The patient stated that the mass had grown suddenly for about 3 months, maybe after a trauma, increasing in volume exponentially and causing him discomfort, embarrassment, and pain. The result of the first diagnostic approach, with the US, was unexpected and suspicious, and the radiologist wanted to do first a CT, and then maybe plan an MRI. The CT revealed an inhomogeneous density formation and in MRI the mass resulted to be compatible, with the radiologic pattern, with the diagnosis of a sarcoma of the soft tissue. The physicians had already alerted the pathological anatomy, as they suspected something malignant. So, some days after the MRI examination, the patient underwent histological sampling, confirming the suspicion: a myxofibrosarcoma (stage III) of the posterior region of the femoral region. The patient started on radio and chemotherapy, which increases survival and in the hope of reducing the size of the mass, and a strict follow-up was posed before doing the surgery.

## Introduction

Myxoid-appearing soft-tissue lesions comprise both benign and malignant entities. Because of the high-water content, these lesions may look as cysts on radiographs, and histological features overlap. In this setting, malignant entities include myxoid liposarcoma, myxoid leiomyosarcoma, myxoid chondrosarcoma, ossifying fibromyxoid tumor, and myxofibrosarcoma [1].

Myxofibrosarcoma is also known as a myxoid variant of malignant fibrous histiocytoma (MFH); it is a type of cancer that begins in the connective tissue. It is one of the most common types of sarcomas affecting older adults.

Myxofibrosarcoma is classified as a type of fibroblastic/myofibroblastic tumor.

This tumor typically appears as a slow-growing, painless mass. Larger tumors may cause pain due to pressure on surrounding tissue.

Surgery is the mainstay of sarcoma treatment: the resection might be extensive, but the recurrence is possible and frequent. Radiotherapy can be utilized as a neoadjuvant or adjuvant treatment to increase local control rates, while chemotherapy is used to shrink large masses before surgery [2].

This case report describes the imaging examination, especially US, CT scans, and MRI in the diagnostic process of a young and unaware patient who experienced pain and discomfort in the posterior region of the right thigh.

## Case report

A 58-year-old patient came to the Radiology Department for an ambulatory diagnostic examination after he expressed discomfort to his primary care physician.

The patient stated that for 3 months he had already been experiencing the exponential growth of an expansive formation in the posterior region of his femoral region, which caused him great discomfort and embarrassment but not pain until that moment.

**Fig. 1:**
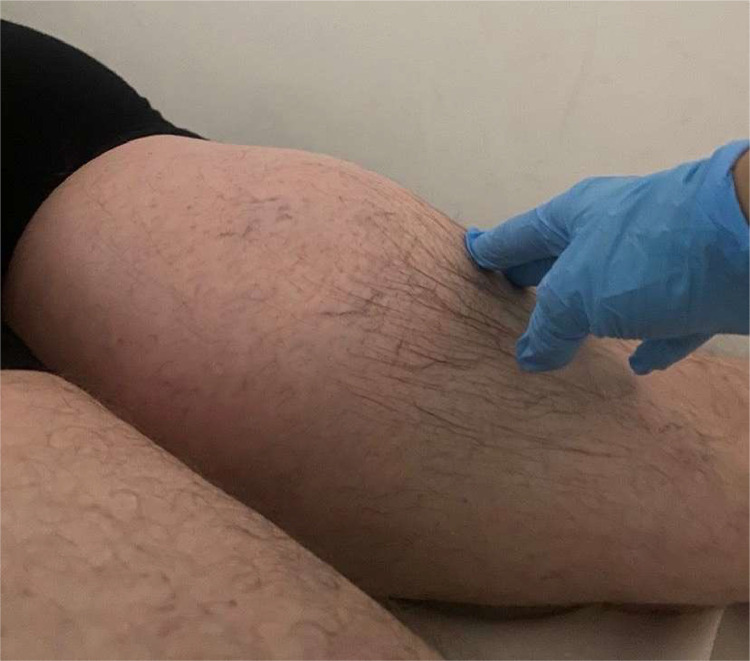
Macroscopic photo of the patient's posterior thigh region on clinical examination: it was visible how an underlying round formation curves the skin surface. The skin appears intact, only affected by some little telangiectasias. The round formation was very hard to touch.

The formation was round, hard to touch, and not very mobile about the underlying planes and had, according to the patient, grown exponentially very rapidly in the last 40 days (Fig. 1).

**Fig. 2:**
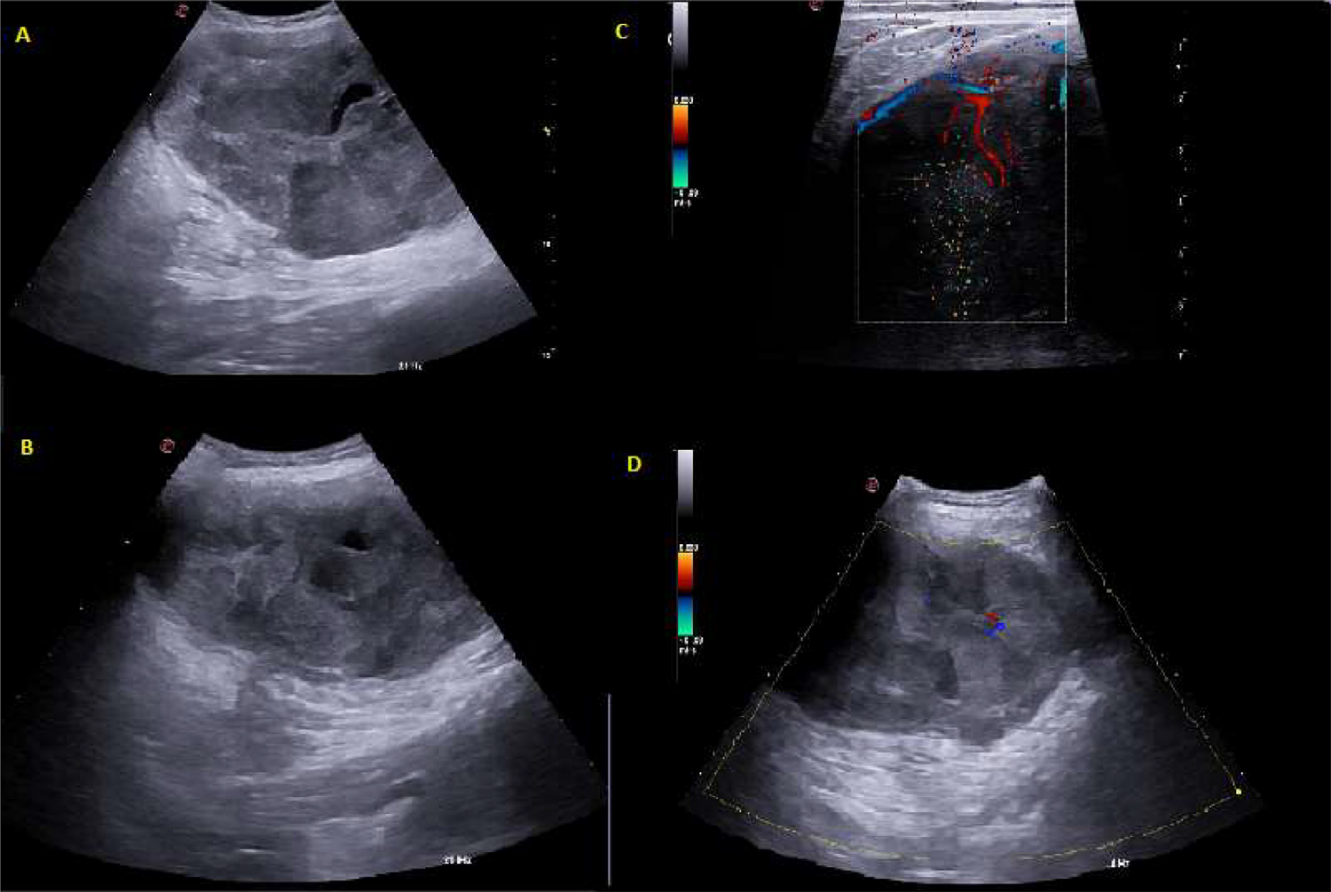
(A and B) Ultrasonography of the formation appears not well characterized given its large extent; it is very inhomogeneous with hypoechogenic/an-echogenic areas mixed with some septa. (C and D) At Colour-Doppler evaluation the vascular signal appears to increase in its vicinity and inside it.

The physician believed first to perform an ultrasound (US) investigation: due to the suspect of a cystic formation or the indecision between something malignant (such as a soft tissue mass) or benign nature; however, the sustained growth rate and little mobility to the fascial plane do not depict anything good. The radiologist proceeded with a US (Fig. 2).

At US examination, the formation appears extremely inhomogeneous, with disorganized hypo-an-echogenic areas: these lesions may appear as cysts but were justified by their high-water content [3].

**Fig. 3:**
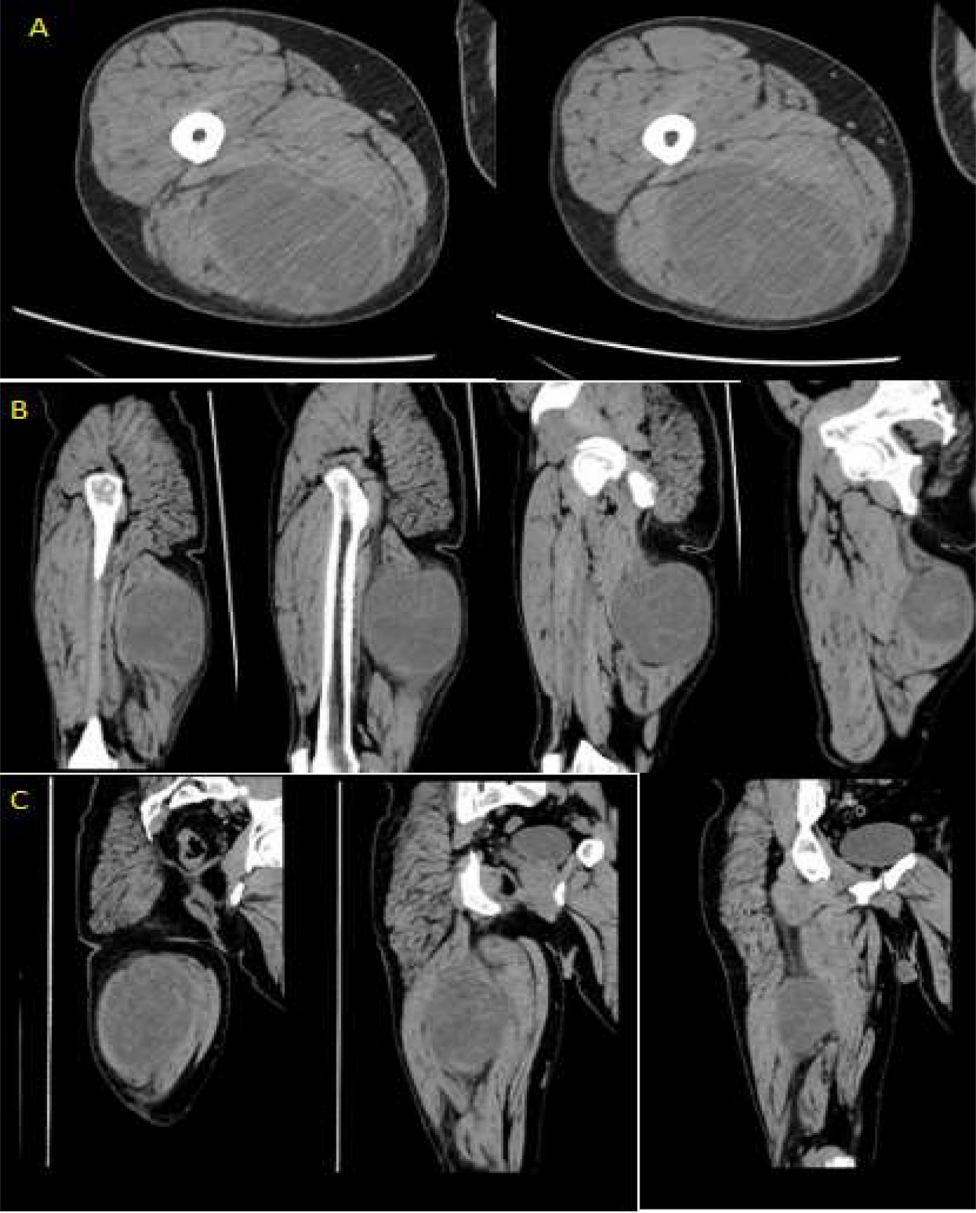
CT-scan without contrast medium of the formation which measures about 28 × 22 cm, in (A) axial plane (B) sagittal plane (C) coronal plane; The subcutaneous formation, from the soft tissue, appears a large lobulated and, relatively well-circumscribed.

The patient, especially after the US examination, complained of pain in the pressure of the probe and due to the initial suspicion of bone involvement, underwent a CT of the femur to better characterize the round and expansive formation and the tissue that surrounds it (Fig. 3).

At CT, the round formation appeared smooth, with a tiny capsule and apparently in the context of the semitendinosus muscle causing mass effect and compression.

**Fig. 4:**
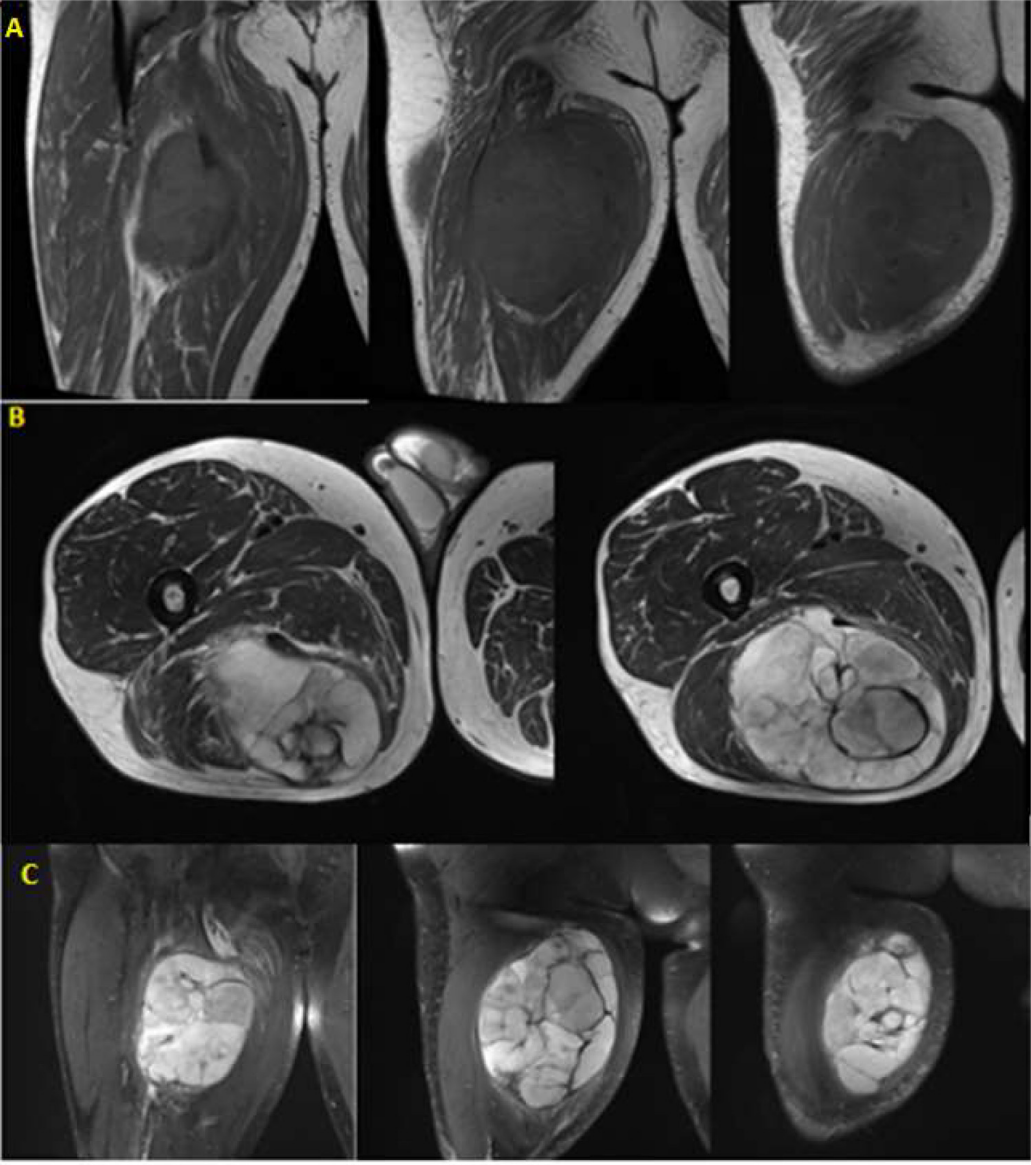
MRI of the posterior region of the right femoral region at different levels of the lesion, in diverse sections (A) coronal plane in T1 (isointense signal); (B) axial plane in T2 (hyperintense signal); (C) coronal plane in pd (proton density) where it appears hyperintense too.

The density was inhomogeneous: the suspicion of something malignant was raised and an MRI was scheduled for the next day, but without contrast medium due to the high creatinine value (Fig. 4).

**Fig. 5:**
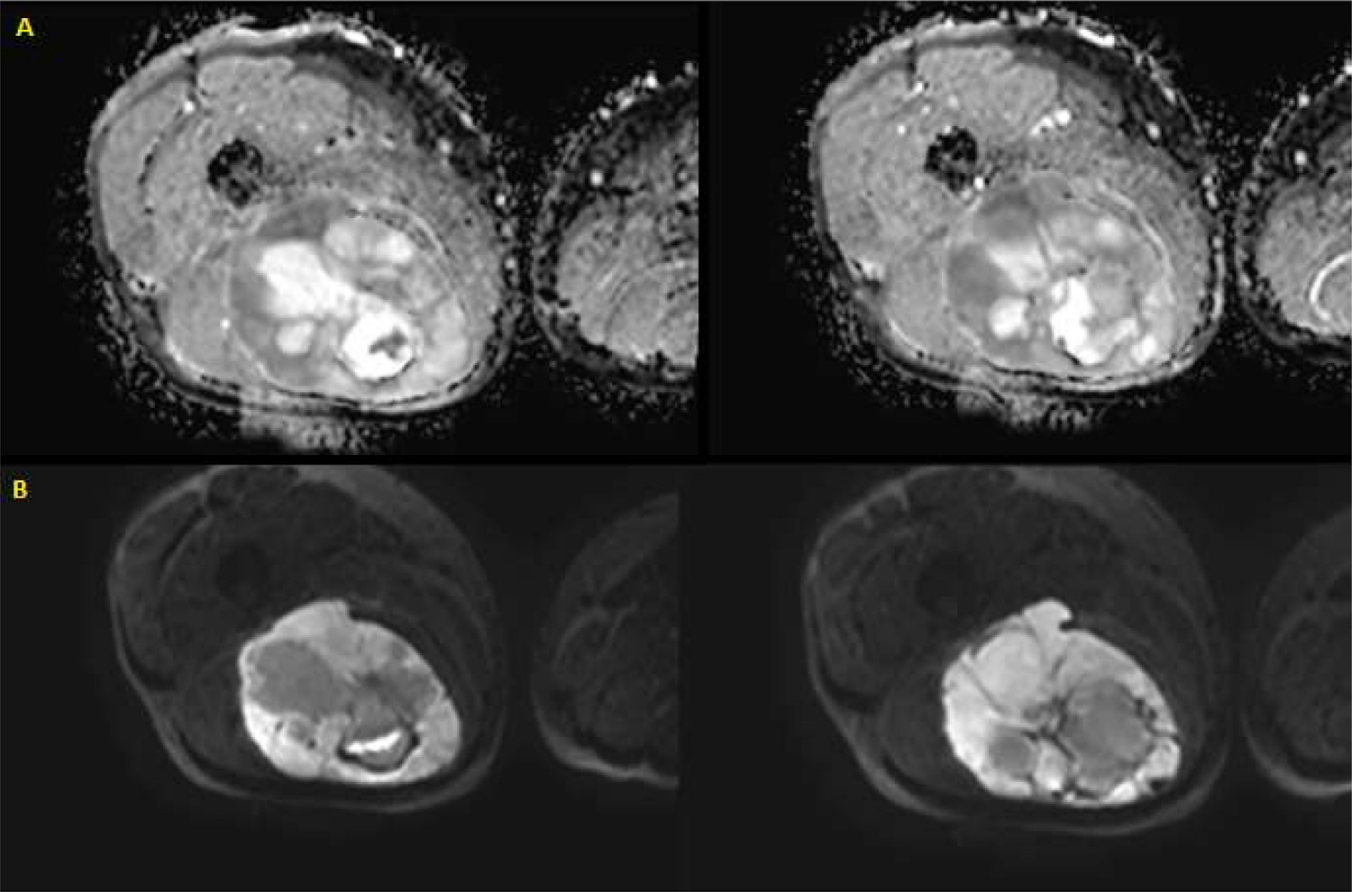
MRI of the posterior region of the right thigh: (A) in the axial section in DWI (diffusion-weighted) at the major level of B-value (s/mm^2^); (B) in the axial section ADC-map; those 2 couple of images are at different levels of the lesion. There were different areas of high cellularity, narrowing in diffusion and confirming the suspicion of malignancy.

At MRI the lesion appeared lobulated and with an inhomogeneous signal: isointense in T1 and hyperintense in T2, STIR, and PD [4]. Focal central areas, with an isointense signal, represent the necrotic or cystic portion. In this case, there wasn't visible the typical, and pathognomonic “fascia tail sign” (Fig. 5).

The radiologist confirmed the suspect: the mass was something malignant, a soft-tissue sarcoma, localized, and the histological test was important to differentiate the tumor.

After some days, the excisional biopsy of the mass was performed, and the result was a myxofibrosarcoma of high grade (Grade 3 according to the FNCLCC system).

Before the excisional surgery, which was required and planned after some weeks/months, the patient underwent a cycle of radiotherapy to increase local control rates; the patient also ensured chemotherapy to shrink the large masses before surgery.

The patient is now being closely monitored while waiting for the operation, despite the fact that such masses have a high incidence of local recurrence even after extensive surgery.

## Discussion

Myxofibrosarcoma, also known as a myxoid variant of malignant fibrous histiocytoma (MFH), is a type of cancer that begins in the connective tissue, characterized by a locally infiltrative activity and an extremely high rate of local recurrence.

This type of cancer is part of soft-tissue sarcomas: a group of tumors where malignant cells are concentrated on such districts as muscles, adipose, blood or lymphatic vessels, ligaments, connective tissues, or nerves. Fifty percent of such disease states tend to affect the limbs, both upper and lower, while the other half of cases predominantly affect the neck and head, abdominal cavity, internal organs, and trunk.

Sarcomas in general, and soft tissue sarcomas in particular, are rather rare cancers [5].

The diagnosis is challenging because patients with myxofibrosarcoma frequently report a painless growing soft tissue tumor in their extremities: in the appearance of a few conspicuous swellings, which tend to enlarge in the short span of a few weeks and only sometimes result in peculiar painful manifestations, due especially to the compression over the surrounding tissue [6], [7], [8].

Sarcomas and soft tissue tumors in general are treatable illnesses in the majority of instances, with the prognosis mostly determined by tumor size, tissue depth, and the presence or absence of metastatic disease. This is because the treatment option of choice has always been surgery: the more radical this can be, the better the chances of defeating the disease; it is important to proceed with surgery with chemotherapy, especially for patients with sarcoma at high risk of recurrence like myxofibrosarcoma [[9], [10]]. The primary treatment for localized illness is surgical excision plus (neo)adjuvant radiotherapy and chemotherapy [[8], [11]]. Tumor size is important because tumors smaller than 5 cm are less likely to spread to other parts of the body and are associated with a better prognosis. Tumor size is also used to determine the pathological stage of the tumor (pT). Pathologists divide myxofibrosarcoma into three grades based on a system created by the French Federation of Sarcoma Group Cancer Centers (FNCLCC).

This approach determines tumor grade using 3 microscopic features: differentiation, mitotic count, and necrosis. This approach classifies myxofibrosarcoma as either a low-grade or high-grade tumor. The grade of the tumor is important because high-grade tumors (grades 2 and 3) are more likely to regrow after surgery and metastasize (spread) to other parts of the body [11].

## Conclusions

Soft tissue sarcomas are extremely rare cancers; myxofibrosarcoma, part of this heterogeneous group, is a type of cancer that begins in the connective tissue.

It is one of the most common types of sarcomas affecting older adults and it is characterized by a locally infiltrative activity and an extremely high rate of local recurrence. The diagnosis is very challenging due to the different types of presentation and the radiologists should all know the radiological findings shown in this case, to raise the suspicion of this specific type of tumor, confirmed only by 1 excisional biopsy in its histology.

## Declarations

Ethical approval No ethics committee approval was sought for this ex vivo animal cadaveric study. No human subject was involved in this study.

## Authors Contribution

All Authors have contributed to the final manuscript.

## Patient consent

Complete written informed consent was obtained from the patient for the publication of this study and accompanying images.
